# Resorption of the calcium phosphate layer on S53P4 bioactive glass by osteoclasts

**DOI:** 10.1007/s10856-019-6295-x

**Published:** 2019-08-14

**Authors:** Nicole A. P. van Gestel, Gerke H. Schuiringa, Juul H. P. H. Hennissen, Anneke C. A. Delsing, Keita Ito, Bert van Rietbergen, Jacobus J. Arts, Sandra Hofmann

**Affiliations:** 1 Orthopaedic Biomechanics, Department of Biomedical Engineering, Eindhoven University of Technology, PO Box 513, 5600 MB Eindhoven, The Netherlands; 2 Institute for Complex Molecular Systems, Eindhoven University of Technology, PO Box 513, 5600 MB Eindhoven, The Netherlands; 3 Faculty Bèta Sciences and Technology, Zuyd University of Applied Sciences, PO Box 550, 6400 AN Heerlen, The Netherlands; 4 Department of Orthopaedic Surgery, Research School CAPHRI, Maastricht University Medical Centre, PO Box 5800, 6229 HX Maastricht, The Netherlands; 5 Department of the Built Environment, Building Physics and Services, Eindhoven University of Technology, PO Box 513, 5600 MB Eindhoven, The Netherlands; 6 Department of Orthopaedics, University Medical Center Utrecht, PO Box 85500, 3508 GA Utrecht, The Netherlands

## Abstract

Clinically, S53P4 bioactive glass (BAG) has shown very promising results in bone infection treatment, but it is also known to degrade very slowly in vivo. To evaluate which mechanisms (cellular or dissolution) can play a role in the degradation of S53P4 BAG and S53P4 BAG putty, in vitro degradation experiments at different pH (7.4 and 4.6) were performed. Micro computed tomography showed a rapid dissolution of the synthetic binder in the putty formulation, within 12 h is simulated body fluid (pH = 7.4), leaving behind only loose granules. Therefore the degradation of the loose granules was investigated further. Significant weight loss was observed and ion chromatography showed that Ca^2+^, Na^+^ and PO_4_^3−^ ions were released from S54P4 BAG granules in the two fluids. It was observed that the weight loss and ion release were increased when the pH of the fluid was decreased to 4.6. Osteoclasts are known to create such a low pH when resorbing bone and therefore their capacity to degrade S53P4 surfaces were studied as well. Scanning electron microscopy and energy-dispersive X-ray spectroscopy confirmed that osteoclasts were able to create resorption pits in the calcium phosphate layer on S53P4 BAG surfaces. The silica of the BAG, located underneath the calcium phosphate, seemed to hinder further osteclastic resorption of the material. To our knowledge we were the first to observe actively resorbing osteoclasts on S53P4 bioactive glass surfaces, in vitro. Future research is needed to define the specific role osteoclasts play in the degradation of BAG in vivo.

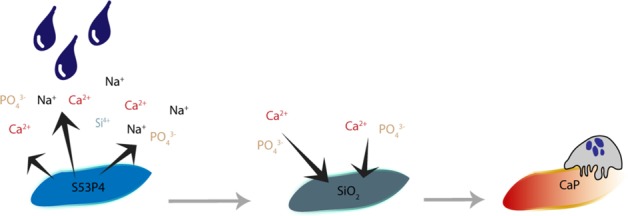

## Introduction

Bioactive glass (BAG) granules with the specific S53P4 composition (53 wt% SiO_2_, 20 wt% CaO, 4 wt% P_2_O_5_, and 23 wt% Na_2_O) have been shown to be able to treat bone infections (osteomyelitis) with high success rates over 90%, because of their antibacterial properties [1–7]. Clinical outcome showed proper infection eradication, but also revealed a very slow in vivo degradation of the material [1–7]. In several cases, granules were still observed in large bone defects more than ten years after implantation [8]. This slow degradation may hinder the full regeneration of the bone, which also has been indicated by a long-term follow-up study that showed a thickened cortex in several patients treated with BAG [8]. In combination with the presence of BAG granules, this may lead to an altered load distribution through the adjacent bone [9, 10]. As many of the cases that may benefit from treatment with BAG are in load-bearing sites, it might become a problem if the bone cannot fully regenerate [2, 8, 11, 12].

It is generally accepted that contact between BAG materials and (body) fluid evokes a release of ions from the BAG, but little is known about (other) mechanisms that are involved in the degradation of BAG materials [1, 13–15]. Wilson and coworkers (2006) showed that osteoclasts were able to attach to the S53P4 BAG surface, but did not observe active osteoclastic resorption of the material [16]. Others found inhibition of osteoclastic development by soluble silica and 45S5 BAG [17]. On the other hand, it has been reported that BAG materials can be dissolved and that this dissolution is faster when the pH of the solution is decreased [14, 18, 19]. As osteoclasts are known to dissolve hydroxyapatite (HA) by decreasing the pH underneath their ruffled borders, it is suggested that osteoclasts not only attach, but can also play a role in the degradation and remodeling of BAG [13, 20].

Earlier studies on the degenerative capacity of osteoclasts on BAG surfaces, did not include the layer formation cascade upon contact with (body) fluid [1, 13–15]. This layer formation is suggested to happen as follows: the contact of BAG with fluid initiates an ion exchange (Na^+^, Ca^2+^, PO_4_^3−^, Si^4+^) with the surrounding fluid. This gives rise to the antibacterial properties by increasing the local pH (creating a local alkaline environment) and osmotic pressure [1, 21–25]. The ion exchange also initiates a silica layer formation on the BAG surface onto which calcium and phosphate then precipitate. This calcium phosphate can crystalize into natural HA over time, which enables bone bonding [1, 14, 15, 26]. This cascade of reactions is instantaneous and the calcium phosphate layer is developed within hours to days [26].

To improve the handling for a surgeon in an operation theatre, an injectable putty formulation was developed [12]. In this putty, S53P4 BAG granules are contained in a binder that consists of polyethylene glycol (PEG) and glycerol, thus forming an extra layer around the BAG. The water soluble binder is thought to dissolve quickly upon contact with body fluid after which the residual granules will fill the defect only loosely, depending on the amount of binder used [12]. Presently, however, it is not clear to what extent the binder will remain and if it can affect the BAG degradation properties.

This study focuses on the passive (pH dependent) and active (osteoclast activity dependent) degradation process of S53P4 BAG with binder in vitro. Our hypothesis is that the binder will dissolve quickly after which degradation of S53P4 BAG is initiated by the release of ions into fluid, but as soon as a calcium phosphate layer is formed on the BAG surface, the continuation of the dissolution is prevented and cellular activity is needed to degrade the material further. As osteoclasts are able to resorb calcium phosphates and HA materials, it is expected that they are also able to resorb at least the calcium phosphate layer on BAG surfaces after contact with fluids [13].

## Materials and methods

### Longitudinal µCT and image analysis of BAG putty dissolution in SBF

Kokubo’s simulated body fluid (SBF) was used in the dissolution experiments of the putty, and was created as previously reported [27]. Briefly, 8.035 g L^−1^ sodium chloride (Merck Millipore, Burlington, Massachusetts, USA), 0.355 g L^−1^ sodium hydrogen carbonate (Merck Millipore), 0.225 g L^−1^ potassium chloride (Sigma Aldrich, Saint Louis, Missouri, USA), 0.231 g L^−1^ potassium phosphate dibasic trihydrate (Merck Millipore), 0.311 g L^−1^ magnesium chloride hexahydrate (Sigma Aldrich), 38 mL 1 M hydrochloric acid (Sigma Aldrich), 0.386 g L^−1^ calcium chloride dehydrate (VWR chemicals, Radnor, Pennsylvania, USA), 0.072 g L^−1^ sodium sulphate (Sigma Aldrich) and 6.118 g L^−1^ Tris (Merck Millipore) was dissolved in deionized water and the pH was adjusted to 7.4 [27, 28].

BAG putty with 21 wt% synthetic binder consisting of PEG and glycerol (BonAlive® Biomaterials Ltd., Turku, Finland), as described in van Gestel et al. (2017), was placed in a bioreactor designed for longitudinal µCT imaging (*n* = 5) [12]. This bioreactor allowed for nondestructive imaging over time without the need for removal the samples from the bioreactors and without scanning artifacts arising from the bioreactor itself [29, 30]. The used bioreactors were spinner flasks bioreactors such as those previously used to monitor ECM formation by mesenchymal stromal cells (MSCs) over time [31]. In the current study, the magnetic stirrer bar was not turning, resulting in no flow/mechanical loading. Imaging was performed prior to SBF addition (*t* = −1), directly after fluid addition (*t* = 0), and three and twelve hours later (*t* = 3 and t = 12) with a µCT scanner (µCT80, SCANCO Medical AG, Brüttisellen, Switzerland). The scan parameters used for µCT imaging were an isotropic voxel size of 36 µm, a voltage of 70 kVp, a tube current of 114 µA, 500 projections per 180°, and an integration time of 300 ms. All samples were incubated at 37 °C with 5% CO_2_ between subsequent µCT scans.

The volume loss of the synthetic binder was determined with image analysis software (IPLFE v02.01, Scanco Medical AG). The binder of the putty was segmented from the µCT images based on the attenuation coefficients by using a threshold of 288–808 mgHA ccm^−1^ (defined as binder volume, BV). To overcome the partial volume effect at the borders of the glass granules in the putty, a two-voxel erosion followed by a two voxel dilatation was applied. The amount of segmented voxels (that represented the BV) was then converted into a volume in cubic millimeters. The volume of the total putty (BAG granules including hte binder) was determined by the segmentation of the complete material (referred as total sample volume, TSV) with a threshold of 288-max mgHA ccm^−1^ at *t* = −1. This way the BV/TSV could be determined. As a control for the image analysis method, five independent samples with only BAG granules (no binder) were scanned and analyzed in the same way resulting in BV = 0 mm^3^, indicating that the glass granules did not interfere with the calculated BV.

### Ion chromatography and weight loss of BAG granules in physiological and acidic buffer solutions

To determine ion release by BAG granules in an in vitro setup, 100 mg S53P4 BAG granules (2.0–3.15 mm, BonAlive®) were submerged in 50 mL Tris/HCl at pH 7.4 or acetate buffer at pH 4.6. Tris/HCl buffer was prepared by adding of 0.2 M Tris, dissolved in deionized water, to 0.2 M hydrochloric acid (Sigma Aldrich) in deionized water, and adjusted to a buffer solution with a physiological pH of 7.4. The acetate buffer was prepared by adding of 0.2 M potassium acetate (Sigma Aldrich) dissolved in deionized water, to 0.2 M acetic acid, resulting in an acidic buffer solution at pH 4.6. The pH was monitored during the experimental period.

The concentration of the released ions in the buffers was examined with ion chromatography (Dionex 1100, Thermo Fisher Inc., Waltham, Massachusetts, USA) 24 h, 72 h and 7 days after immersion. In addition, the weight loss of these granules was determined at these time points after drying the samples in air. The fluid was not refreshed during the experimental period.

Na^+^ and Ca^2+^ concentrations were measured using an ion exchange column CS12A (2 × 250 mm) with 20 mM methasulfonic acid as eluent and an isocratic flow of 0.25 ml min^−1^. Detection of ions was done by suppressed conductivity (Dionex CSRS 500 2 mm). Na^+^ IC standard (Aldrich 43492) and Ca^2+^ IC standard (Aldrich 39865) were used for calibration. The concentration of anions (phosphate (PO_4_^3−^)) was analyzed using a Dionex 1100 with an AS9-HS (2 × 250 mm) ion exchange column, with 9 mM sodium carbonate (Na_2_CO_3_) as eluent and an isocratic flow of 0.25 ml min^−1^. Detection of ions was done by suppressed conductivity (Dionex AERS 500 2 mm). Calibration was done with the anion mix IC standard (Aldrich 89886). All experiments were performed with *n* = 3 and ion chromatography quantifications were performed by the external standard method.

### Monocyte isolation and osteoclastic differentiation

For cell culture experiments human peripheral blood mononuclear cells (hPBMCs) were isolated from buffy coats from a healthy donor (Sanquin Blood Supply Foundation, Nijmegen, The Netherlands). The isolated cells were mixed with citrate buffer (6 g sodium citrate in 1000 mL phosphate buffered saline (PBS, Sigma Aldrich)) and centrifuged (800 g) in an isosmotic medium with a density of 1.077 g mL^−1^ (Lymphoprep™, STEMCELL Technologies Inc, Vancouver, British Columbia, Canada). Monocytes were subsequently isolated from the obtained hPBMCs by magnetic active cell sorting, according to the manufacture’s protocol (Monocyte isolation kit II, Miltenyi Biotec, Bergisch Gladbach, Germany). The undifferentiated monocytes were seeded at a density of 3.0 × 10^5^ cells per cm^2^ in the wells of a 24 wells plate to which a Transwell® insert (polyethylene membrane, 6.5 mm, 3.0 µm, Corning Inc., Corning, New York, USA) containing 100 mg S53P4 BAG granules (2.0–3.15 mm, 53 wt% SiO_2_, 20 wt% CaO, 4 wt% P_2_O_5_, and 23 wt% Na_2_O, BonAlive® Biomaterials Ltd.) was added. This way, the ions were released into the cell culture medium of the monocytes. The monocytes were cultured in 5% CO_2_ at 37 °C in RPMI 1640 medium (Gibco, Thermo Fisher Inc.) supplemented with 10% fetal bovine serum (FBS, Greiner Bio one, Kremsmünster, Austria) and 1% Penicillin/Streptomycin (Lonza, Basel, Switzerland). To promote osteoclastic differentiation, the cells were primed with 50 ng mL^−1^ macrophage-colony stimulating factor (M-CSF, Peprotech Inc, Rocky Hill, New Jersey, USA) during the first two days. After those two days of priming, the medium was supplemented with 50 ng mL^−1^ M-CSF and 50 ng mL^−1^ receptor activator of nuclear factor (NF)-κB (RANKL, Peprotech Inc.) for the full culture period [32]. Medium was changed every 2–3 days. At each medium change, the BAG granules were substituted with 100 mg fresh granules to assure ion release during the whole culture period. As a control, cells were cultured in the same conditions but without the addition of BAG. After 14 days of culture, immunohistochemistry was performed to determine osteoclastic differentiation by TRAP expression staining. Briefly, cells were fixed with 3.7% formaldehyde (Merck Millipore) in PBS and permeabilized with 0.5% Triton X-100 (Merck Millipore). Non-specific binding was blocked by 10% horse serum (Invitrogen, Carlsbad, California, USA) in NET-gel (which consisted of 50 mM Tris, 150 mM sodium chloride (Merck Millipore), 5 mM ethylenediaminetetraacetic acid (EDTA, Sigma Aldrich), 0.05% nonidet P-40 substitute (Fluka, Sigma Aldrich) and 0.25% gelatin (Sigma Aldrich)). TRAP was labelled with a goat anti-human primary antibody (Santa Cruz, Dallas, Texas, USA) and stained with a secondary Alexa 488 donkey anti-goat antibody (Molecular probes, Eugene, Oregon, USA). Actin filaments and cell nuclei were counter stained with TRITC-conjugated Phalloidin (Sigma Aldrich) and DAPI (Sigma Aldrich), respectively. Samples were visualized using an inverted microscope (Zeiss Axiovert 200 M, Carl Zeiss Microscopy, Jena, Germany). For quantification of the cell size of the TRAP positive, multinucleated cells (osteoclasts), the area enclosed by the actin ring was measured using ImageJ (National Institutes of Health, Bethesda, Maryland, USA). In addition, the number of nuclei per osteoclast (DAPI) was counted.

Undifferentiated monocytes of the same batch of hPBMCs were also cultured directly on BAG discs (*n* = 7, 53 wt% SiO_2_, 20 wt% CaO, 4 wt% P_2_O_5_, and 23 wt% Na_2_O) with a rounded shape of and size of 13 mm by 13 mm and a thickness of 1.5 mm (BonAlive® Biomaterials Ltd). Prior to cell seeding, the discs were secured in polydimethylsiloxane (PDMS consisting of 10:1 w/v parts base to curing agent (Sylgard 184, Dow Chemical Company, Midland, Michigan, USA)) in 24 wells plates. These secured plates with their surface exposed were presoaked in PBS for three days for the formation of a calcium phosphate layer. As a control, the monocytes were seeded on dense HA discs (*n* = 8) with a diameter of 9.5 mm (Himed Inc., Old Bethpage, New York, USA), also secured in PDMS. Cell seeding density was 3.0 × 10^5^ cells per cm^2^, and cells were cultured up to 18 days, with the same conditions as mentioned before. After the culture period, examination of the resorption activity of the osteoclasts was performed using SEM and EDX.

### SEM and EDX

SEM (Quanta 600, FEI, Thermo Fisher Inc.) was performed to examine the surfaces of granules soaked in Tris/HCl and acetate buffers, and to evaluate the morphology of the cells and resorption pits on the surfaces of the BAG and HA discs. Samples without cells were dried in air for at least 24 h, while the samples with cells were fixed with 2.5% buffered glutaraldehyde (10% glutaraldehyde (Sigma Aldrich) in 0.1 M sodium cacodylate buffer (Sigma Aldrich)), after 15 days of culture (*n* = 4). These cell-containing samples were dehydrated using alcohol series and chemically dried with hexamethyldisilazane (Sigma Aldrich). To examine resorption pit formation, cells were removed with 10% household bleach after 18 days of culturing (*n* = 3) [33]. After three washing steps with water, the samples were dried by air. All samples, with cells and with removed cells, were sputter coated with 5 nm gold (Q300TD, Quorum Technologies, Laughton, United Kingdom). The formed resorption pits were quantified by measuring the resorbed surface area per pit, this was performed by one observer with the ImageJ software. The resorbed area per pit was assessed for 16 and 17 resorption pits found on BAG and HA discs, respectively.

EDX (EDAX Genesis 2 system 60 for Quanta 600) was performed together with SEM, to analyze the elements present at the sample surfaces and in the resorption pits. By color-coding the specific elements, maps of the elements on the surface of BAG were generated.

### Statistical analysis

Statistical analysis was performed with R (packages: Rcmdr, PMCMR, and PMCMRplus). For all statistical tests an α-level of 0.05 was used for significance, all data is expressed as mean ± standard deviation. A Student T-test was performed to detect differences between two groups and ANOVA to detect significant differences when more than two groups were involved. When ANOVA was indicated to be significant, a post hoc test was applied with a Holm correction (R default). In case of a sample size *n* < 5, or when normality (Shapiro-Wilk test), or equal variances (Levene’s test) could not be assumed, nonparametric tests were conducted. Wilcoxon was used as an alternative for the T-test and Kruskal-Wallis as an alternative for ANOVA. The Dunnett T3 correction in post hoc analysis was used when a Kruskal-Wallis test was applied because of unequal variances, in case of equal variances a Conover correction was used in post hoc analysis.

## Results

### Dissolution of the synthetic binder in a BAG putty material

**Fig. 1:**
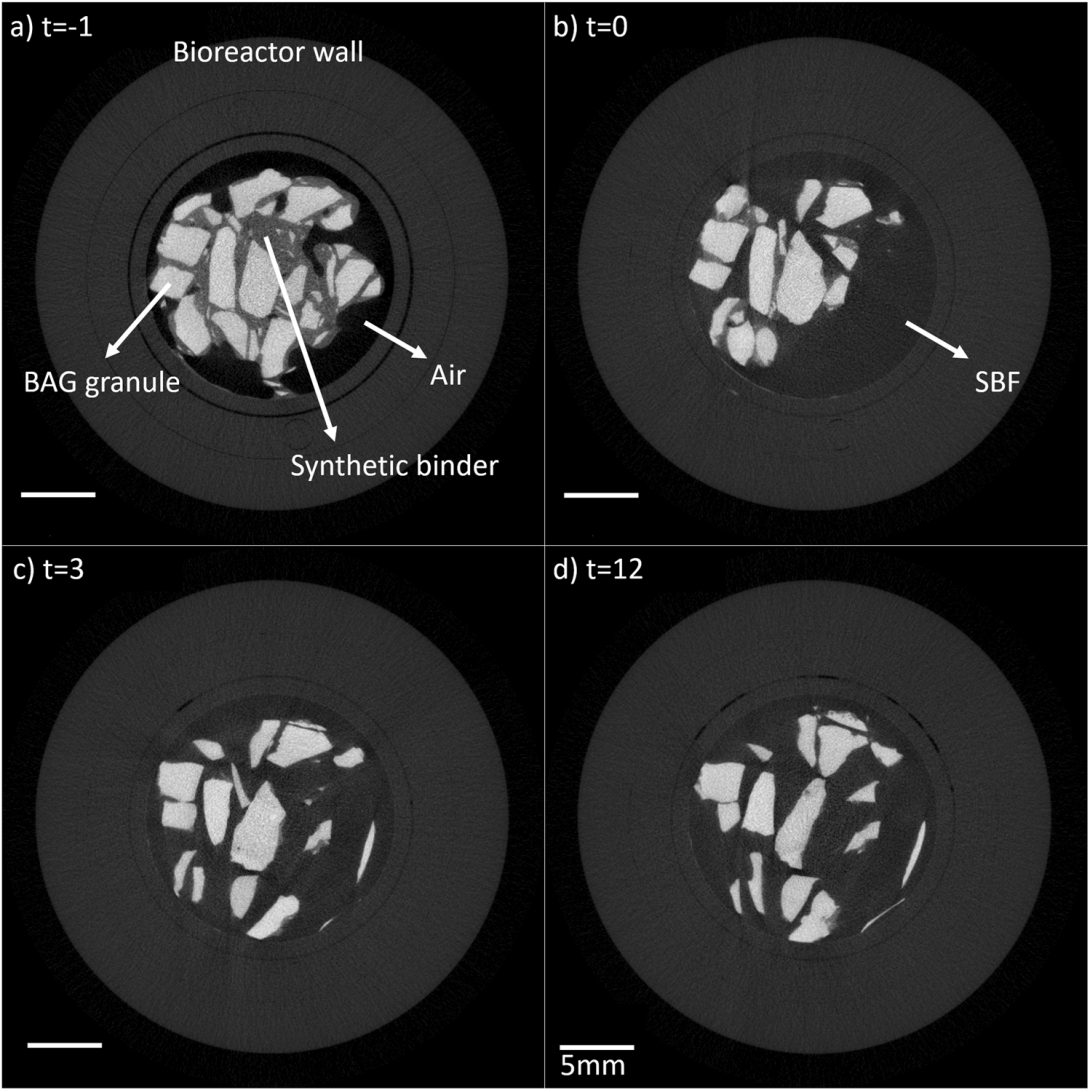
Grayscale images (before segmentation) of one BAG putty sample that was submerged in SBF at *t* = 0. Images of the putty sample were obtained **a** prior to the addition of SBF (*t* = −1), **b** directly after the addition (*t* = 0), **c** 3 h after the SBF addition (*t* = 3) and **d** 12 h after SBF addition (*t* = 12). In between the scans, the samples were kept at 37 °C

**Fig. 2:**
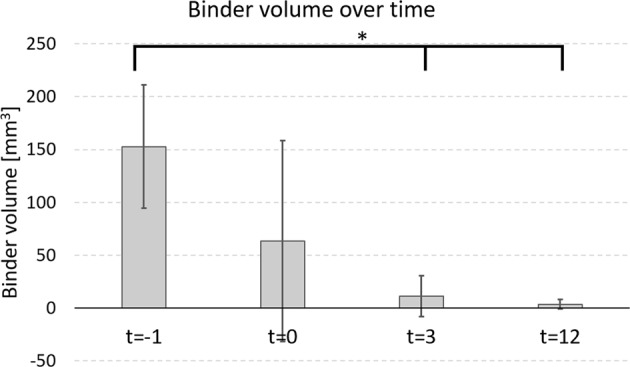
The putty binder volume over time as observed with µCT image analysis. The initial binder volume at *t* = −1 was lost within hours, due to SBF addition at *t* = 0. Significance is indicated at * a *p*-value < 0.05

To determine the dissolution of the PEG and glycerol containing binder in a BAG putty, it was longitudinally imaged with µCT while submerged in SBF, and the binder volume was determined at several time points (Fig. 1). In the µCT greyscale images, all putty components can be distinguished based on their attenuation coefficients (Fig. 1a, b). The dissolution of the binder started directly with the addition of the SBF at *t* = 0 (Fig. 2) and statistical differences were observed between the volume at *t* = −1 and at *t* = 3 (*p*-value = 0.017) and at *t* = −1 and at *t* = 12 (*p*-value = 0.02). Since Levene’s test indicated unequal variances, a Kruskal-Wallis test with Dunnett T3 post hoc analysis was used to determine the significance. To determine the extent of the binder loss, the volume of the initial binder volume was determined, the BS/TSV at *t* = −1, and this resulted in a volume loss of 0.32 ± 0.06%. As a result of this binder loss, BAG granules were not contained as tight in the holder of the bioreactor and therefore moved, as observed at *t* = 3 and *t* = 12 (Fig. 1c, d).

### Ion release and weight loss by BAG granules in a physiological and acidic environment

**Fig. 3:**
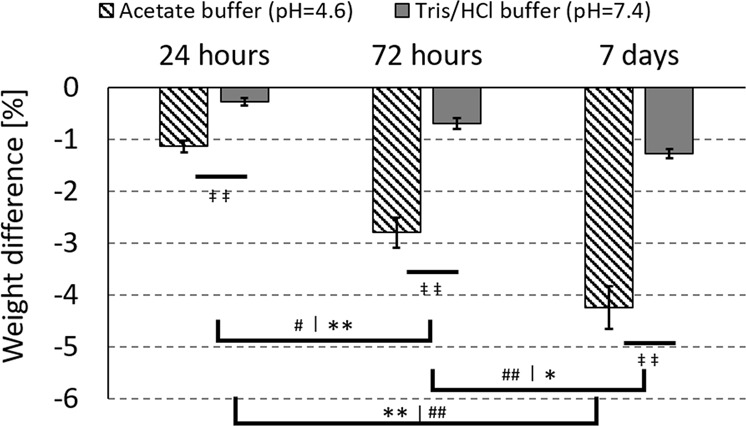
Significant weight loss of the BAG granules in physiological (Tris/HCl) and acidic (acetate) buffers, over time. The * indicates the difference over time in acetate buffer, the # indicates the difference over time in Tris/HCl buffer and the ‡ indicates the difference at the given time point between the two buffers. Single signs refer to a *p*-value < 0.05 and double signs refer to a *p*-value < 0.001

The weight loss of BAG granules submerged in Tris/HCl (pH = 7.4) or acetate buffer (pH = 4.6) was determined and the weight loss over time was significant at both pHs, with a larger weight loss in the acidic buffer compared to the buffer with a physiological pH (Fig. 3), the weight loss was significant between the buffers at 24 h (*p*-value = 0.00028), 72 h (*p*-value = 0.000011) and at 7 days (*p*-value = 0.00028). To determine the significance a Kruskal-Wallis with Conover post hoc analysis was used because of the sample size (*n* = 3).

**Fig. 4:**
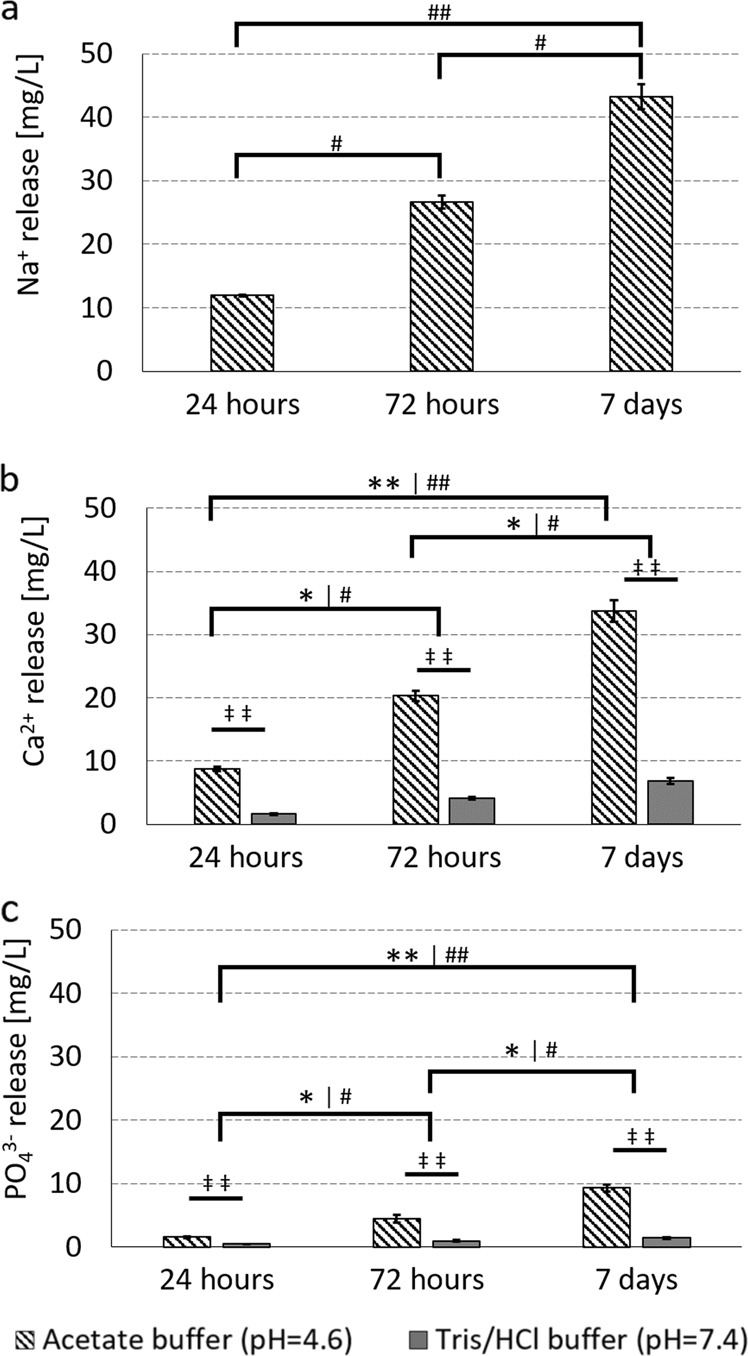
Ion release from BAG at acidic and physiological pH. For **a** sodium, **b** calcium and **c** phosphate. The * indicates the difference over time in acetate buffer, the # indicates the difference over time in Tris/HCl buffer and the ‡ indicates the difference at the given time point between the two buffers. Single signs refer to a *p*-value < 0.05 and double signs refer to a *p*-value < 0.001

The ion release of Na^+^, Ca^2+^ and PO_4_^3−^ was measured with ion chromatography of the buffers in which the BAG granules were submerged. In both buffers, the amount of released ions (mg L^−1^) increased significantly over time (Fig. 4**)**. The results also showed more released Ca^2+^ and PO_4_^3−^ ions in the acidic buffer, with a significant differences between the physiological and acidic buffer at 24 h (Ca^2+^, *p*-value = 0.0000015, PO_4_^3−^ *p*-value = 0.0000575), at 72 h (Ca^2+^, *p*-value = 0.0000015, PO_4_^3−^ *p*-value = 0.0000285) and at 7 days (Ca^2+^, *p*-value = 0.0000015, PO_4_^3−^ *p*-value = 0.0000575). These statistical differences were determined by Kruskal-Wallis with Conover post hoc analysis. The Tris/HCl buffer interfered with the Na^+^ in the ion chromatography measurement, therefore the data of the release of Na^+^ in the Tris/HCl buffer (pH 7.4) is missing in Fig. 4.

### Composition changes on the BAG surfaces in an acidic and physiological environment

**Fig. 5:**
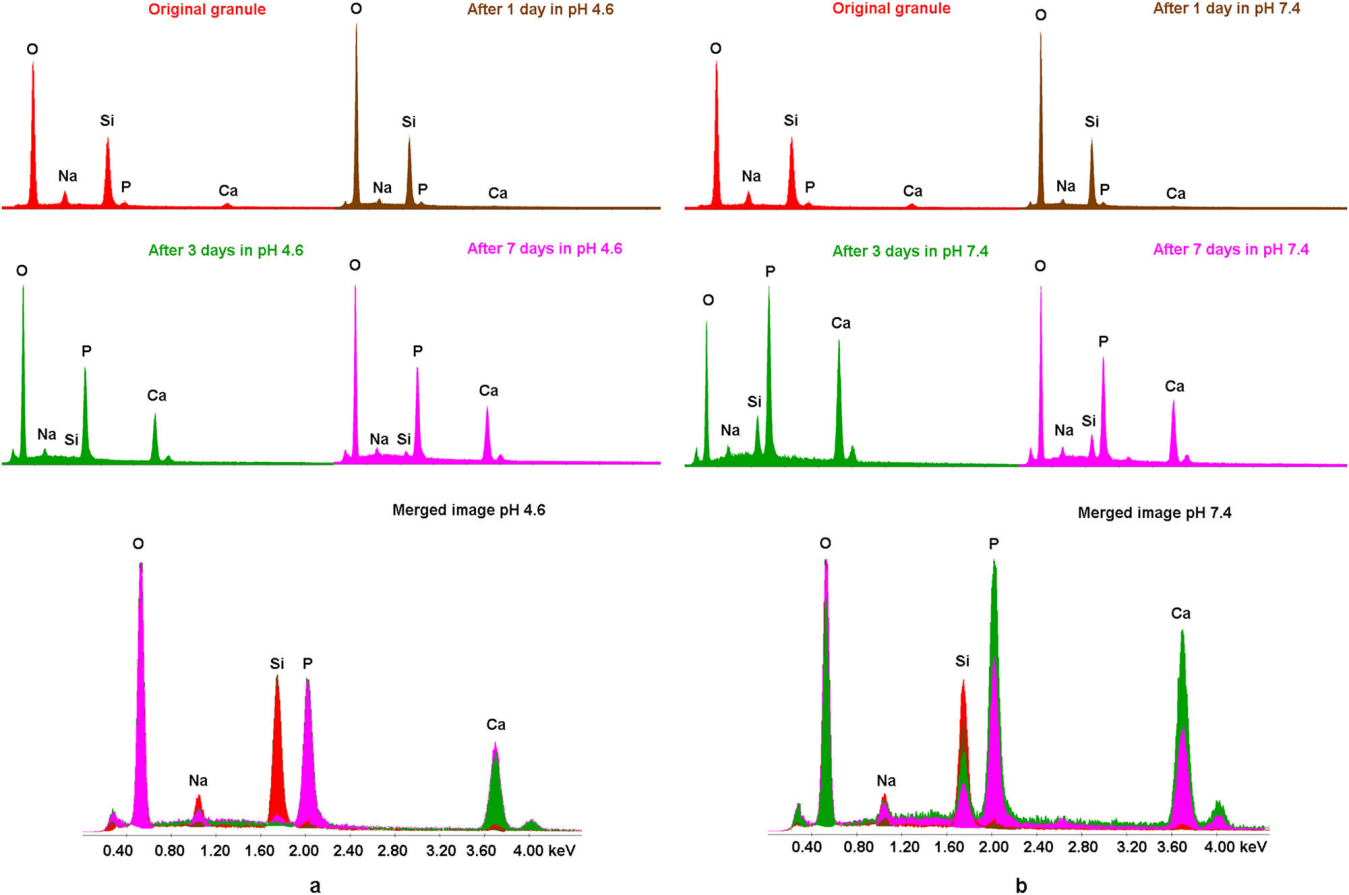
Calcium phosphate precipitated on BAG granule surfaces after three days in physiological and acidic buffer solutions. **a** EDX spectra on BAG granules submerged in acetate buffer (pH = 4.6) and **b** submerged in Tris/HCl buffer (pH = 7.4) up to 7 days

With SEM and EDX the surface composition of an original BAG granules (red line in Fig. 5) was compared to the surface of the composition of granules submerged in an acidic and physiological fluid for 7 days and this showed a clear calcium phosphate layer formation in both groups (Fig. 5). On the samples submerged in Tris/HCl buffer at pH 7.4, a clear decay of silica on the BAG surface could be observed over time. This decay is less pronounced on the BAG that was submerged in the acetate buffer solution at pH 4.6. Initially, no calcium and phosphate were observed on the BAG surface, but it clearly had precipitated after 3 days in both solutions (Fig. 5).

### Osteoclastic differentiation of human monocytes in the presence of BAG

**Fig. 6:**
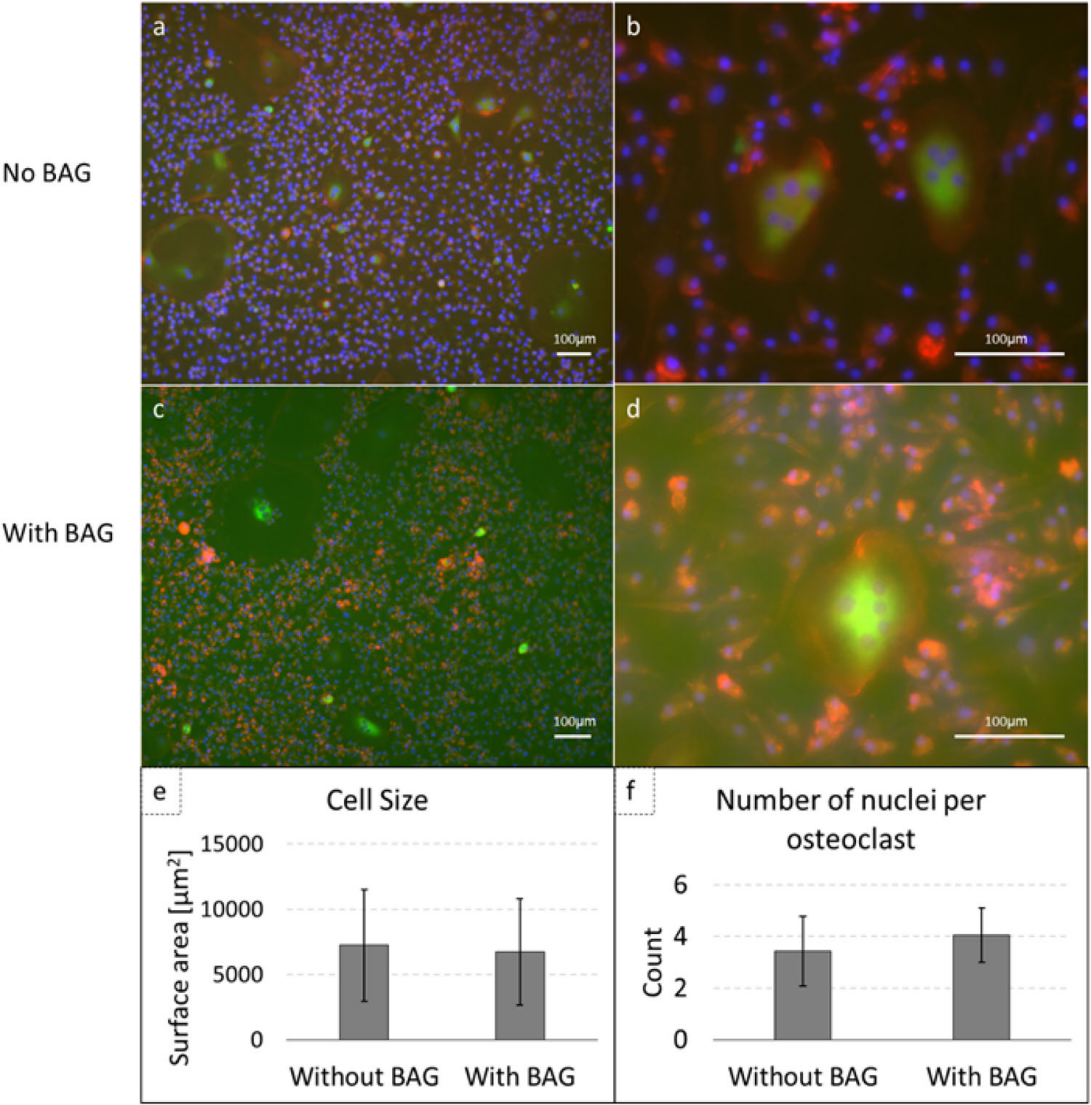
Osteoclastic differentiation of human monocytes is not hampered by the presence of BAG. **a**, **b** Immunofluorescent images of human monocytes cultured on culture plastic (control), **c**, **d** immunofluorescent images of human monocytes on culture plastic in presence of BAG. TRAP is stained in green, nuclei in blue (DAPI) and actin is stained in red (Phalloidin-TRITC). **e** Cell size per osteoclast. **f** Number of nuclei per osteoclast

Monocytes were successfully isolated from hPBMCs from human buffy coats. After differentiation with M-CSF and RANKL either in the presence or absence of BAG, immunofluorescent stained cells were visualized using fluorescence microscopy (Fig. 6**)**. In both cultures, multinucleated, TRAP expressing cells with prominent actin rings were observed, which is typical for osteoclasts. Similar numbers of osteoclasts were observed in both cultures and the cell size and number of nuclei per osteoclast were counted (*n* = 18 with BAG and *n* = 14 without BAG). No significant differences were observed in cell size (6725.0 ± 4086.1 µm^2^) or number of nuclei per osteoclast (4.1 ± 1.1 counts), when compared to controls that were not exposed to BAG (respectively, 7236.3 ± 4294.9 µm^2^ and 3.4 ± 1.3 counts) (Fig. 6e, f). For statistical analysis a Wilcoxon rank sum test was performed as normality could not be assumed according to the Shapiro-Wilk test.

### Osteoclastic resorption on pre-soaked BAG surfaces

**Fig. 7:**
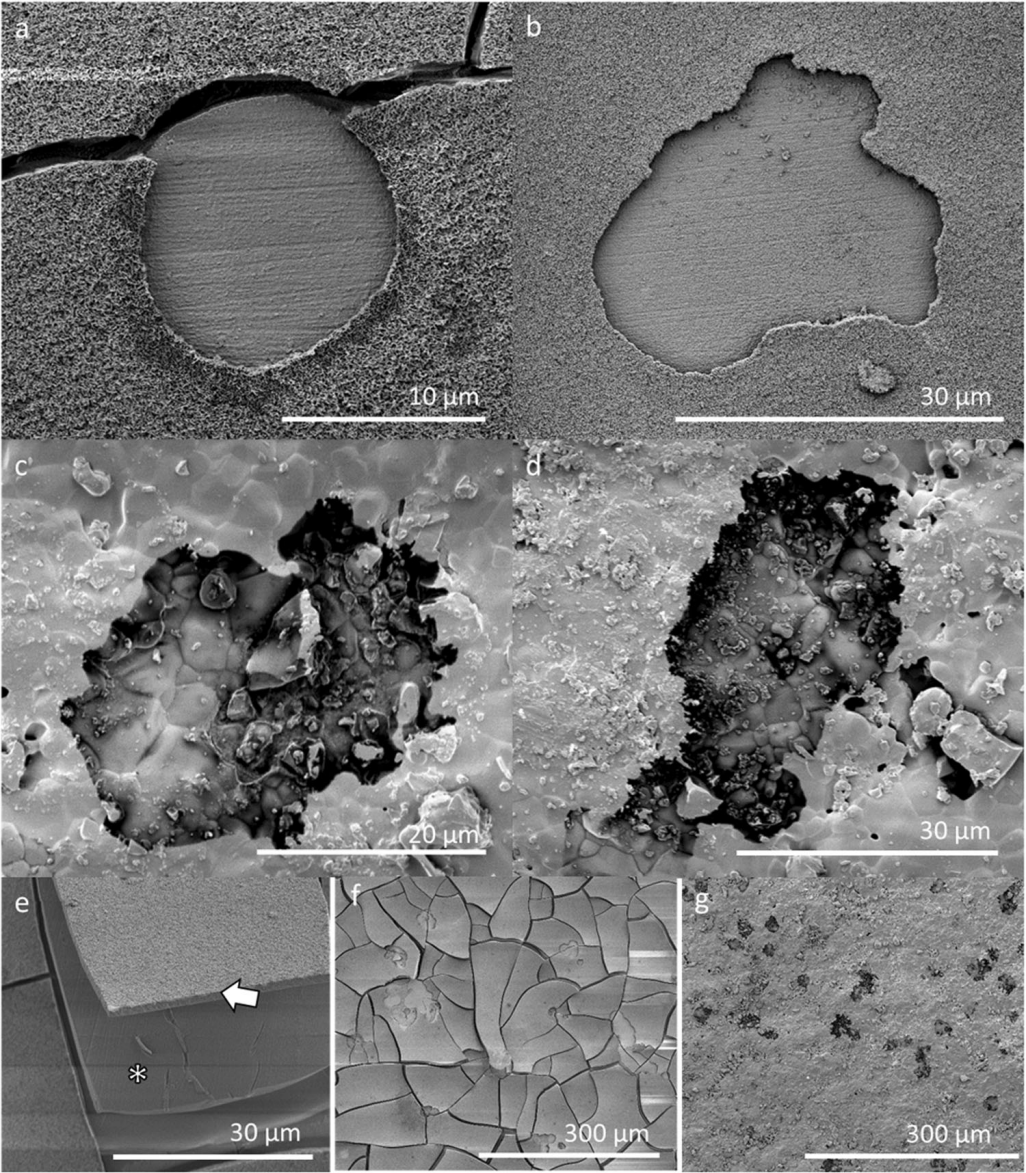
Osteoclastic resorption of BAG and dense HA surfaces (**a**, **b**) on BAG discs, the pits show a very smooth and uniform surface. **c**, **d** on dense HA discs, with a less smooth and uniform surface. **e** Layer of calcium phosphate (arrow) that exposed the silica layer (*) underneath. **f** Overview of the resorption pits found on a BAG surface and on (**g**) a HA surface

**Fig. 8:**
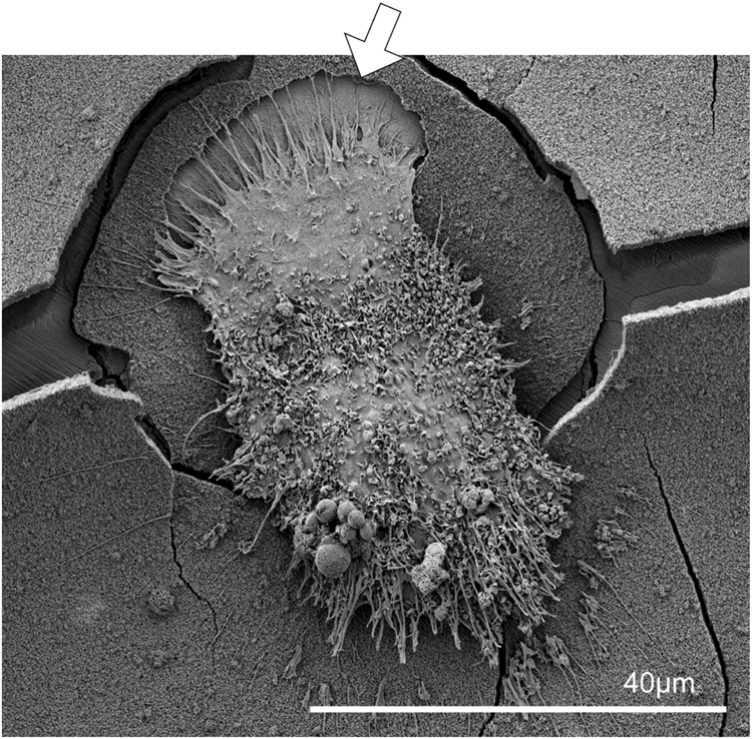
Close-up of an osteoclast that is moving away from a resorption pit (arrow) on a BAG disc

Calcium phosphate precipitation had taken place before the addition of cells (3 days of pre-soaking in PBS prior to cell seeding) and this was confirmed with SEM and EDX (Fig. 7). The cracks in the BAG samples are probably caused by dehydration of the samples during sample preparation for SEM analysis. The osteoclasts were able to attach to the calcium phosphate layer on the BAG surface and were able to actively resorb it (Figs. 7 and 8). In some samples the calcium phosphate layer seemed to detach from the silica layer (Fig. 7e), which was confirmed by EDX analysis (data not shown). The appearance of the resorption pits seemed to be different on both surfaces (Fig. 7). The resorption pits on BAG had a very smooth and uniform appearance, while the pits on HA were rough. The area of the resorption pits was measured on both surfaces and these results show that the pits found on HA were significantly larger than pits found on BAG (*p*-value = 0.0011, determined with the Wilcoxon rank sum test).

**Fig. 9:**
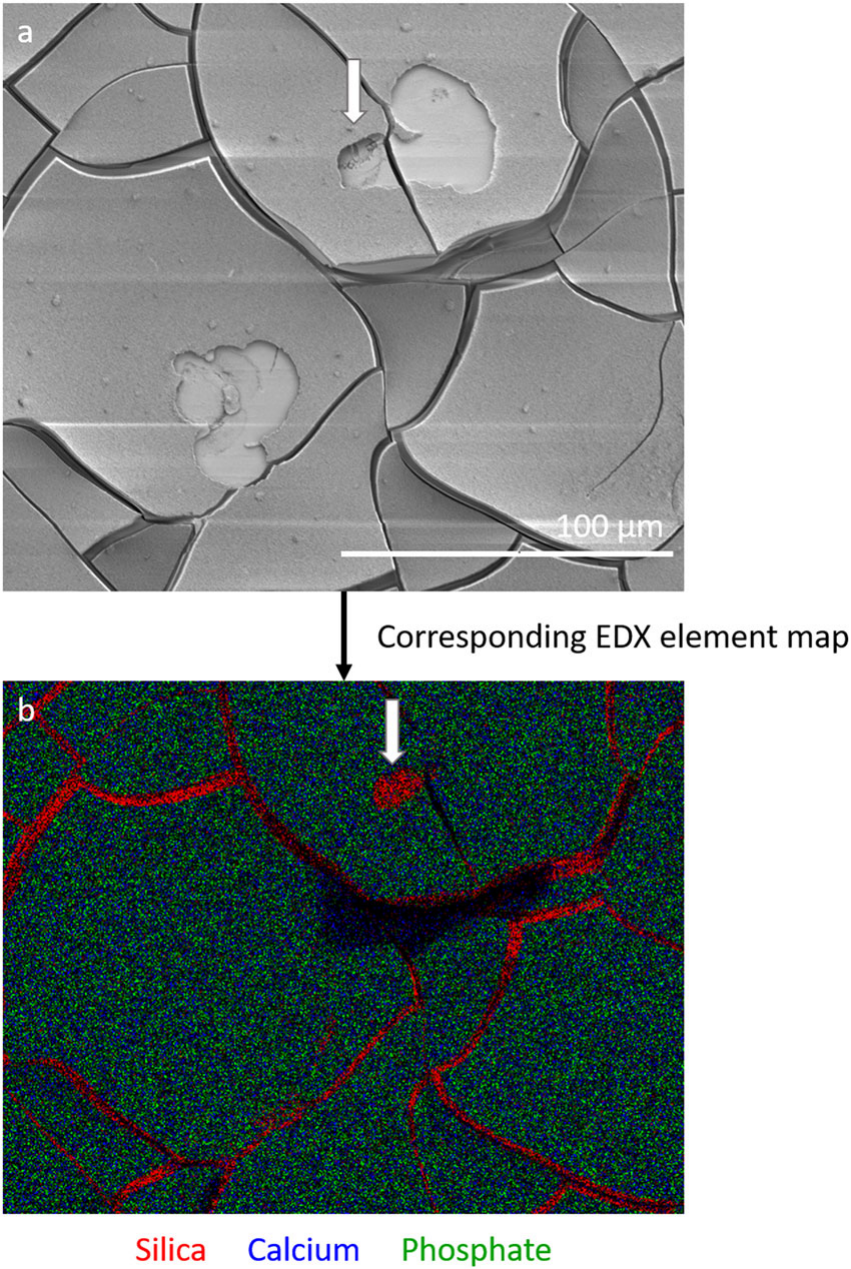
Resorption by osteoclasts did not completely remove the calcium phosphate layer on the BAG surface. **a** A SEM image of two resorption pits on a BAG disc surface, **b** the associated EDX map of the BAG disc showing silica in red, calcium in blue and phosphate in green. Silica is detected in the cracks and only in a small part of the resorption pit (white arrow). Most of the surface consisted of calcium and phosphate

EDX was used to determine the elementary composition of the BAG surface as well as on the exposed surface within the resorption pits (Fig. 9). The main elements detected in the resorption pits were calcium and phosphate. In overview of the element maps also silica is observed in the cracks of the calcium and phosphate layer.

## Discussion

This study focused on the passive and active degradation processes of BAG with and without binder in vitro, to study osteoclastic resorption. The loose granules were degraded faster in an acidic environment and it was observed that osteoclasts that can create such an acidic environment could actively resorb the calcium phosphate on the BAG surface. To the knowledge of the authors, this is the first time that actively resorbing human osteoclasts were observed on BAG. Not only degradation of the loose S53P4 BAG granules was studied, but also the dissolution of a synthetic binder that is incorporated in a putty to create better handling properties.

The results obtained in this study showed that the synthetic binder in the BAG putty material dissolved quickly (within 12 h) in SBF. This binder was added to the granules to create an injectable and better to handle material, the putty. Directly after the addition of the SBF, the dissolution of this binder started. This is reflected by the big standard deviation of the samples at *t* = 0 compared to *t* = −1, as the addition of the fluid distorted the samples. This distortion was also determined by the image analysis algorithm and resulted in this wide range of results. After 3 h, this distortion disappeared. More than 30% of the volume of the complete BAG putty material (BAG + binder) was gone after 12 h. This volume loss is substantial and assuming a similar dissolution time in vivo (or at least in order of magnitude) this would lead to undesired dead space in a bone defect as the granules will collapse. This was also observed in our in vitro results, where granules had moved after the SBF had dissolved the binder (Fig. 1). Therefore, the volume loss, due to binder dissolution, increases the risk for mechanical failure, which makes the material inappropriate for load-bearing applications. In addition, although it has not yet been evaluated whether the BAG putty composition has equal antibacterial properties as S53P4 without binder, the space arising this quickly after implantation potentially also could cause problems concerning reinfection in the treatment of osteomyelitis. As incomplete filling of the defect during the treatment of osteomyelitis has been described to be the cause of reinfections previously [7].

To investigate the influence of the pH on the degradation of the loose granules that are left after binder dissolution, both an acidic and a physiological environment was created by using two buffer solutions. A pH of 4.6 was used to mimic the acidic environment underneath the ruffled border of an osteoclast [20, 34]. A physiological pH was used as a control. The acetate and Tris/HCl buffers were chosen because these have been reported to be stable at the desired pH, 4.6 and 7.4 respectively, and do not contain any of the ions of interest. In both buffers the pH was monitored and stayed constant over the 7 days. The weight loss results and ion release profile is in correspondence with previously reported studies [14, 18, 19, 35]. Because it is expected that only little Si^4+^ is released in the surrounding fluid, its presence in the fluids was not evaluated with ion chromatography. Previous studies have reported that most of the silica stays in the material and forms the silica rich layer underneath the calcium phosphate [14, 15, 19, 36].

The finding that acidic acetate buffer (pH 4.6) dissolved the BAG faster than the Tris/HCl (pH 7.4), suggested that osteoclasts that can create such a low pH underneath their ruffled borders, should theoretically be able to dissolve BAG in this way [20]. Although, actively resorbing osteoclasts were observed on the BAG surfaces, but it is suggested that the BAG was only resorbed by the osteoclast partially as mainly calcium and phosphate were found on the surfaces in the pits (after cell removal). This calcium phosphate also could have precipitated right after the osteoclasts moved away from the resorbed area, or during sample preparation for SEM and EDX, as fluids are involved which could lead to leakage of ions and precipitation of calcium and phosphate. However, in addition the smooth and uniform appearance of the surface within the resorption pits on BAG, compared to the resorption pits on HA surfaces (Fig. 7), suggests that the resorption was at some point hindered in the BAG samples. A possible reason for this hindrance might have been the reported silica layer underneath the calcium phosphate layer. With these findings it remains unclear whether the osteoclasts evoke a new release of ions from the BAG that was covered by the calcium and phosphate [13]. The suggestion that silica hinders the osteoclastic resorption corresponds with the previous findings by Wilson et al. [16]. In that study, rat bone marrow that contained osteoclasts were seeded directly on S53P4 BAG plates, without pre-soaking. Although they observed TRAP expressing cells, they did not observe resorption pits [16]. The reason for this difference might have been the pre-soaking and subsequently created calcium and phosphate layer in our study, or because the TRAP positive cells stained in the study by Wilson et al. (2006) were not foreign body giant cells rather than osteoclasts [37]. The monocytes seeded in the current study, were able to differentiate into multinucleated, TRAP positive, actively resorbing cells and are therefore true osteoclasts [38]. This differentiation previously had not been observed and was described as being inhibited by 45S5 BAG [17, 39]. These differences might be caused by the amount of released silica, as it has been described that the response is dose dependent [33]. The 45S5 composition initially releases more silica than the S53P4 composition, and in addition that morphology, size and shape may play a role in silica release dosage [14, 15, 19, 36]. Care has to be taken when directly comparing different glass compositions, as the difference in spatio-temporal release of ions might have a direct effect on cells in a dose-dependent manner [40]. In addition, the ion release profile and calcium phosphate precipitation are dependent on the available surface of the materials, which too has to be taken into account [41].

Compared to the in vivo situation, in vitro cell experiments have their limitations and can provide only a simplified, but more controlled, environment. In our experiments, only one cell donor was investigated, while it is known that results may vary between donors. It is known that in vivo, bone cells (e.g. osteoclasts, osteoblasts, osteocytes) effect each other’s activity and behavior [38, 42]. Therefore co-cultures could be considered to understand the cellular processes around BAGs better. With this simple in vitro setup we could show for the first time that human osteoclasts are able to degrade BAG surfaces. However, it should be kept in mind that the in vitro setup may not fully resemble the in vivo situation as it has been reported that in vitro an in vivo experiments correlate poorly [43]. It had already been reported that the in vivo degradation in slow, the reasons for that may not become clear from these in vitro experiments. One explanation for the slow in vivo degradation might be that osteoclasts cannot get, or are not triggered to migrate, to the center of the graft. Leaving the center intact and only play a role in the degradation of the construct at the edges. S53P4 BAG is a dense BAG (melt-quenched) and when it is well compacted in a defect, it might impede cellular migration [15, 44]. However, similar dense 45S5 (melt-quenched) showed a slightly faster degradation, both in vitro and in vivo [45]. Incorporating a putty material might change the compactness of the BAG, but as the dissolution of the synthetic binder has shown to be extremely fast (in vitro), it might not be a good alternative due to poor mechanical stability and possibly increased risks for reinfections after dissolution of the synthetic binder in vivo. Cellular degradation of BAG might be enhanced by decreasing the silica content, but such an alteration might also decrease the antibacterial properties of the BAG [23]. For osteomyelitis treatment, antibacterial properties are more important than the degradation rate but if the material is aimed to be used for the treatment of a different (bone) disease, adjustments may be beneficial.

Future research is needed to evaluate the effect of the BAG composition in the (cellular) degradation process. To follow degradation over time, in vivo experiments are needed with longitudinal monitoring of the remodeling of the BAG. In addition, histology is needed to determine if osteoclasts are present in a, with BAG grafted, bone defect and if so, how time plays a role in their presence. In the current study, the bioactive glass discs were presoaked in PBS for three days, prior to cell seeding. A calcium phosphate layer had been formed before the monocytes were added, this precipitation has been described to happen hours to days after the contact with (body) fluid [15]. The pre-soaking approach was used in this study as we expect that in an in vivo situation, osteoclasts have to be recruited after the graft layer has been implanted, therefore; calcium and phosphate could have precipitated before osteoclasts are present at the grafted site [13, 46].

Changing the composition of the BAG might result in faster degradation, but it might also result in inactivation of (resorbing) cells or decreased antibacterial properties [47]. The degradation speed may also be affected by the size and shape of the BAG material, as these determine the free surface area and therefore the ion release pattern [14, 15, 19, 36]. The optimal degradation speed will depend on the application. For the treatment of osteomyelitis, the priority is eradication of the infection. Therefore the slow degradation speed of the granules may not be considered as a major problem [1–7]. For other applications; however, an increased degradation speed may be preferred, e.g. bone regeneration in non-infected load-bearing defects [9]. Future research is needed to study whether the (altered) load distribution due to the fast binder dissolution and the slow degradation of the S53P4 granules is a long-term risk for osteomyelitis patients treated with this specific composition.

## Conclusion

The synthetic binder in BAG putty material dissolves within hours when exposed to fluid. This leaves a substantial empty volume and loose S53P4 BAG granules. The remaining granules release ions resulting in the development of a calcium phosphate layer at their surface. Both, weight loss and ion release, was faster when the pH of the environmental fluid was decreased. Since osteoclasts are able to create such a low pH underneath their ruffled borders to resorb bone, the osteoclastic contribution in the degradation of BAG surface was studied. To the knowledge of the authors, for the first time human monocytes were successfully differentiated in actively resorbing osteoclasts on S53P4 BAG. These osteoclasts were able to resorb parts of the formed calcium phosphate layer on the BAG surfaces. However, resorption of the full thickness of the calcium phosphate layer was not observed. We suggested that the silica layer underneath the calcium phosphate is hindering osteoclasts to further degrade the material, which is a potential mechanism that makes in vivo degradation slow. Changing the composition of the BAG might increase degradation rates, but might also affect the main advantages of the S53P4 composition, namely the antibacterial properties and the bone bonding properties.
